# Clinical and surgical safety in patients transitioning from percutaneous to transcutaneous active bone conduction implants

**DOI:** 10.1007/s00405-025-09610-7

**Published:** 2025-08-08

**Authors:** Leonardo Elías Ordóñez Ordóñez, Gabriela Elizabeth Corredor García, Jorge Medina-Parra, Esther Sofía Angulo Martínez, Silvia Carolina Vanegas, Estefany Catherine Hernández, Maja Magdalena Olsson, Sindi Amaya

**Affiliations:** 1 Department of Otolaryngology, Clinica Universitaria Colombia, Clínicas Colsanitas, Grupo Keralty, Calle 23 #66‑46, Bogota, Colombia; 2 Department of Otolaryngology, Facultad de Medicina, Fundacion Universitaria Sanitas (Unisanitas), Grupo Keralty, Bogota, Colombia; 3 Hospital Militar Central, Universidad Militar Nueva Granada, Bogota, Colombia; 4 Anaboleas Research Team, Endorsed by Clinica Universitaria Colombia and Fundacion Universitaria Sanitas (Unisanitas), Recognized by Colciencias (2024) Ministry of Science, Technology and Innovation of Colombia, Bogota, Colombia; 5 Cochlear Limited, Mölnlycke, Sweden

**Keywords:** Bone anchored hearing aids, Conductive hearing loss, Mixed hearing loss, Surgical outcomes, Follow-up study, Audiological findings, Quality of life

## Abstract

**Purpose:**

The primary aim of this study was to assess clinical and surgical safety for patients who underwent transition surgery from a percutaneous to an active transcutaneous bone conduction implant. A secondary aim was to report audiological changes and outcomes for quality of life post the transition surgery.

**Methods:**

An observational, descriptive and prospective study was performed in a tertiary referral center. Patients who underwent transition surgery as part of their treatment were included. Clinical and surgical safety were assessed through intraoperative and postoperative complications and adverse events. The audiological outcomes were assessed with the Hearing in Noise Test, both fixed noise and adaptative noise measurements. Quality of life changes were evaluated using the Glasgow Benefit Inventory, postoperatively.

**Results:**

The study of 23 cases (18 patients) found minor complications in 5.3% (6/113 follow-up points) and no major complications. Audiometric results showed no significant differences in speech understanding (fixed noise=-0.011%, *p* = 0.99; adaptive noise = 0.236dB, *p* = 0.617). Quality of life scores increased moderately (+ 27.0 ± 33.9), with notable gains in general health (+ 29.9) and social support (+ 31.8), and a modest improvement in physical well-being (+ 10.6).

**Conclusions:**

The study showed that transitioning from a passive percutaneous to an active transcutaneous bone conduction implant is safe, with no major adverse events and only a few minor complications. The audiological outcomes with transcutaneous implants were maintained with respect to percutaneous implants, while quality of life improvements were notable in general health and social support. Clinicians should use these insights to guide patient discussions and tailor post-operative care.

**Supplementary Information:**

The online version contains supplementary material available at 10.1007/s00405-025-09610-7.

## Introduction

Hearing health is acknowledged by the World Health Organization (WHO) as a critical public health concern, with over 1.57 billion individuals worldwide experiencing hearing loss [1, 2]. Hearing loss often adversely affects individuals’ quality of life (QoL) and imposes substantial economic burdens on societies where hearing needs remain unaddressed [1]. Technologies such as hearing aids are widely utilized to improve hearing function in individuals with hearing loss. However, traditional hearing aids often fail to provide adequate benefits or are unsuitable for individuals with certain forms of conductive hearing loss (CHL) and mixed hearing loss (MHL), for example those with microtia or the sequelae of chronic otitis media (COM) [3]. Additionally, some patients with single-sided deafness (SSD) find hearing aids ineffective, necessitating devices that reroute the auditory signal [3].

Bone conduction implants (BCI) offer a viable solution for some patients with CHL, MHL, and SSD. These implants can be either percutaneous or transcutaneous [4]. Percutaneous bone conduction devices transmit sound vibrations directly from the sound processor through the abutment-implant, to the skull bone. In contrast, passive transcutaneous devices utilize a magnetic field to connect the sound processor to the implant, while active transcutaneous devices have an implanted transducer to generate and conduct sound waves through the bone [4, 5].

Percutaneous BCIs provide excellent audiological performance [6], however, they are sometimes associated with skin-related complications that may require medical intervention [4]. In some cases, severe or recurrent skin reactions can necessitate device discontinuation and, in extreme instances, may lead to implant removal or replacement [4]. For patients experiencing complications with percutaneous BCIs, transitioning to a transcutaneous device presents a viable alternative. Among the available BCI options, active transcutaneous implants are particularly advantageous, as they offer superior audiological outcomes compared to passive transcutaneous systems [6]. Consequently, when considering a transition from a percutaneous BCI to a transcutaneous solution, active transcutaneous devices represent the preferred choice. When considering the transition to a transcutaneous device, it is essential to evaluate multiple factors, including clinical safety, auditory performance, and overall QoL. A comprehensive assessment of these parameters can guide healthcare professionals and patients in selecting the most appropriate solution, ensuring an optimal balance between effectiveness and long-term benefits. The primary aim of this study was to assess clinical and surgical safety for patients who underwent transition surgery from a percutaneous to an active transcutaneous BCI. A secondary aim was to report audiological changes and outcomes for QoL post the transition surgery.

## Materials and methods

### Study design and settings

This observational, descriptive, prospective study included patients who underwent surgery to transition from percutaneous to transcutaneous active BCI at a tertiary referral center in Bogotá, Colombia. The transition surgeries were conducted between December 2020 and February 2023, with the data collection concluding in August 2023. The study design and reporting of findings followed the STROBE guidelines, which provide recommendations for the transparent reporting of observational studies [7].

### Inclusion/exclusion criteria

To identify eligible participants, a search was conducted within institutional records to locate patients scheduled for BCI surgery. Patients scheduled for a transition from a percutaneous BCI to an active transcutaneous BCI as part of their treatment were included in the study. Eligible patients under 15 years of age, as well as those with neurological or cognitive impairments, and patients with SSD were included in the clinical safety analysis but excluded from the hearing in noise test and QoL analyses. Audiological performance and QoL were reported exclusively in the subgroup of patients with CHL and MHL to ensure homogeneity within the study population. Due to the small number of SSD cases receiving BCI in our institution, this report does not provide representative outcomes for SSD.

Patients with a history of passive transcutaneous BCI were also excluded from the study due to the distinct surgical procedures compared to those involving percutaneous implants, as well as the anticipated lower incidence of infection or skin-related complications in this group.

### Data collection and outcome variables

A digital form was created to collect comprehensive patient data including demographic, clinical, surgical, and postoperative follow-up information. The primary outcome was clinical and surgical safety (intraoperative and postoperative complications and/or adverse events), evaluated in the entire study group. Secondary outcomes were audiological performance pre- and post-surgery and patient-reported QoL following transition surgery only assessed for patients with CHL and MHL. Data on audiological performance with the percutaneous implant was derived from previous routine audiological assessments (conducted in quiet conditions) documented in the patients’ medical records.

### Sociodemographic and clinical characteristics

Pre-transition data, including demographics, clinical characteristics, usage, and hearing performance with the percutaneous implant, were extracted from patient medical records. Assessing documentation on Holger’s score [8] was deemed particularly important as it evaluates skin reactions at the previous percutaneous implant site, which may influence or be a pre-requisite for the decision to proceed with transition surgery.

### Clinical safety outcomes

Clinical safety outcomes included intraoperative complications and adverse events occurring during the postoperative follow-up period. Major complications were defined as infections at the surgical site, implant extrusion, or the need for revision surgery. Minor complications encompassed hematomas, implant site pain, headaches, seromas requiring needle aspiration, and any other symptoms reported by the patient. Postoperative follow-up evaluations to assess clinical safety were conducted two to five days post-surgery, during sound processor fitting, and at one, three, six, and 12 months, with subsequent assessments every six months until the study’s conclusion.

### Audiological measurements and outcomes

Audiological outcomes were assessed by measuring preoperative air conduction and bone conduction thresholds, as well as postoperative free-field thresholds with the BCI activated. The pure-tone average (PTA) was calculated using four frequencies (0.5–3 kHz), and the air-bone gap (GAP) was determined by subtracting the PTA bone conduction from the PTA air conduction. Postoperative thresholds with the device were measured in a free field setup, with the speaker positioned at 0° azimuth, 1 m from the participant’s head. Functional gain (FG) was calculated by comparing preoperative unaided PTA with postoperative aided PTA. Speech audiometry was conducted to assess the speech recognition threshold (SRT) and the speech discrimination score (SDS) by using disyllabic lists.

Audiological performance in noise was assessed by the Hearing in Noise Test (HINT) [9], conducted both with the percutaneous device (approximately four weeks before transition surgery), and with the transcutaneous device (after three months of device use). Speech recognition percentages were determined by having patients repeat phrases presented in both quiet and noisy conditions [9]. Audiological outcomes were only assessed for patients with CHL/MHL who were of the age 15y and older who did not have any neurological or cognitive impairment, to ensure the reliability of these evaluations.

### Assessment of postoperative QoL

**Fig. 1:**
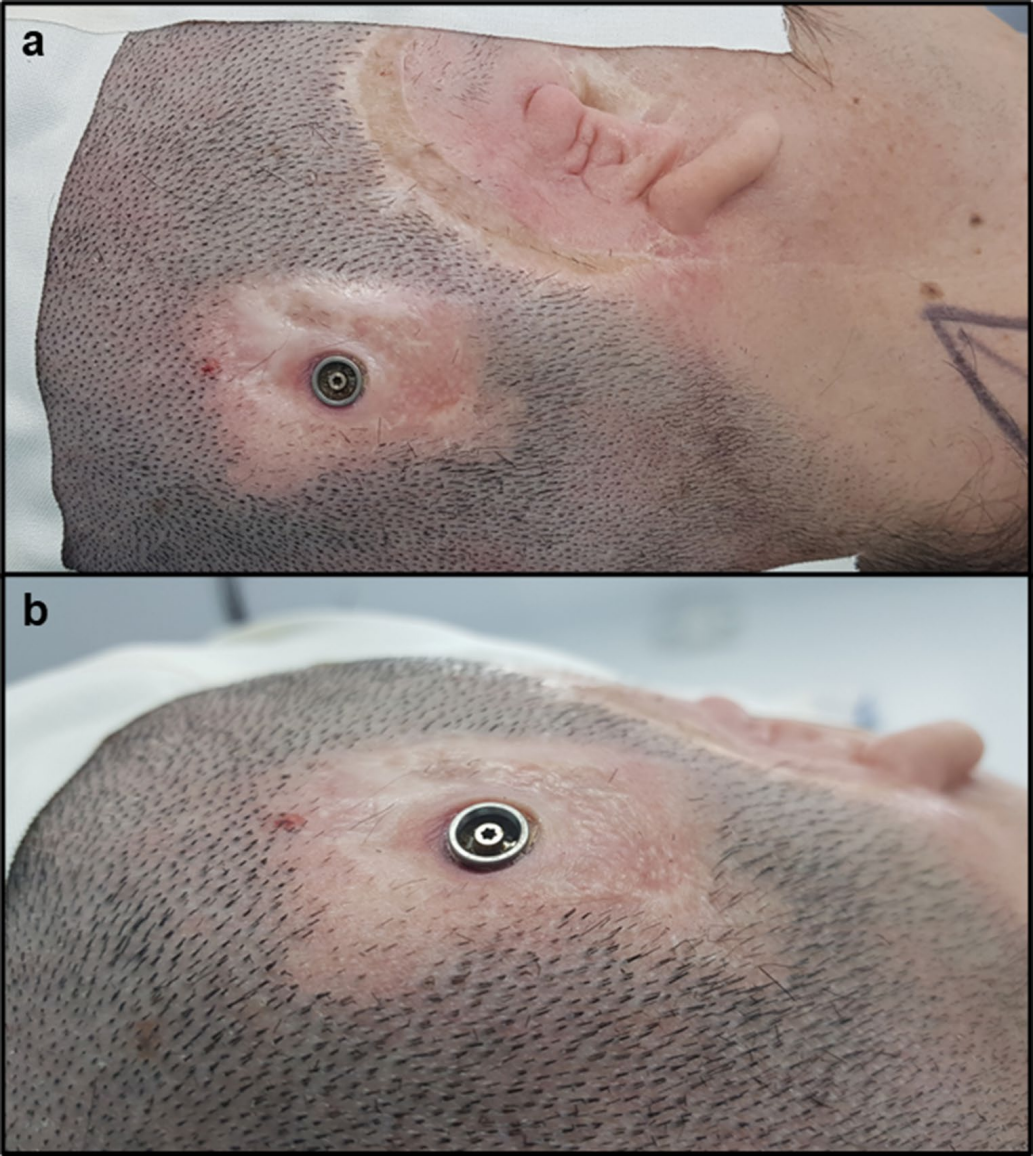
A patient with a percutaneous BCI who required transition to an active transcutaneous BCI due to recurrent skin complications. A patient with microtia and atresia of the right external auditory canal underwent implantation of a percutaneous osseointegrated device using dermatome technique with an inferior pedicled flap. The patient experienced excellent auditory outcomes and used the BCI for 19 years. However, recurrent skin reactions necessitated multiple courses of topical and systemic antibiotics, as well as cutaneous BCIorticosteroid infiltrations. Ultimately, the patient underwent transition surgery to an active transc

Quality of life was assessed postoperatively, after patients had used the transcutaneous device for more than three months, using the Glasgow Benefit Inventory (GBI), which has been validated in Spanish [10]. The GBI is designed to evaluate changes in health and quality of life status following an intervention, such as surgery [10]. The Glasgow Benefit Inventory [11] consists of 18 questions that assess changes in three subcategories: general health status, social support and physical well-being. Responses are rated on a 5-point scale (1 = Much worse, 2 = Somewhat or slightly worse, 3 = No change, 4 = Somewhat or slightly better, and 5 = Much better). The responses are then scaled and averaged to give a score which ranges from − 100 (poorest outcome) through 0 (no change) to + 100 (best outcome). Interpretation of the scores reflects the perceived benefit of the intervention: positive scores (+), indicate a perceived improvement of QoL, a score of 0 indicates no change, and negative scores (-) suggest a perceived decline in QoL following the intervention [11]. Quality of life outcomes were only assessed for patients with CHL/MHL who were of the age 15y and older who did not have any neurological or cognitive impairment, to ensure the reliability and replicability of these evaluations (Fig. 1).

### Surgical technique of the transition surgery

**Fig. 2:**
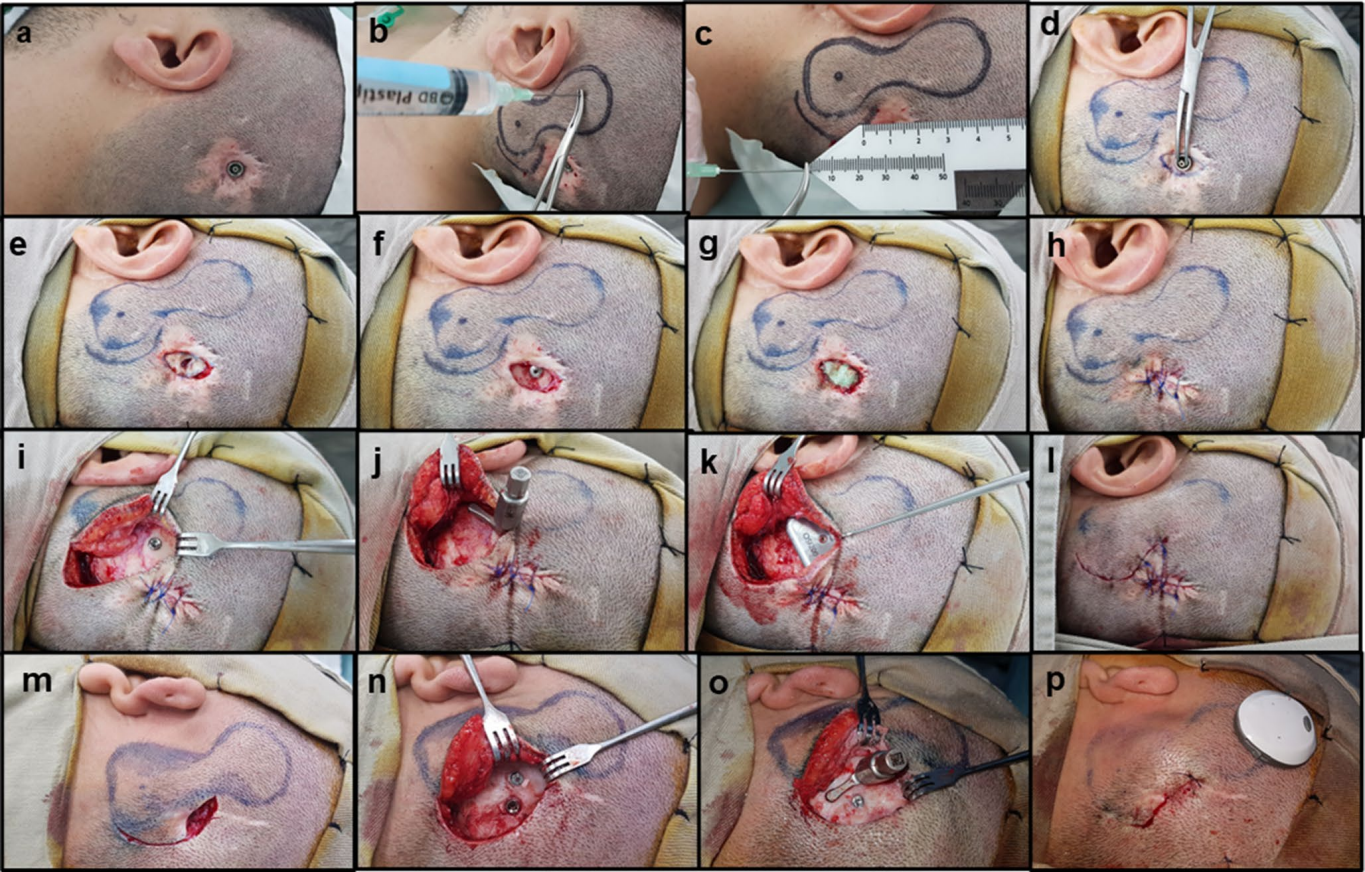
Surgical technique for transitioning from a percutaneous osseointegrated (BAHA Connect™) to an active transcutaneous (Osia™) BCI. The surgical procedure involves several key steps to ensure optimal implant placement and minimize complications: (**a**) Preparation of the surgical site, (**b**, **c**) measurement of flap thickness to ensure optimal coupling and information transmission (recommended thickness ≤ 9 mm), (**d**) removal of the abutment, (**e**) resection of the skin and, (**f**) subcutaneous tissues to minimize bacterial colonies, (**g**) thorough cleaning of the area with chlorhexidine for at least 5 min, limited to the implant site, (**h**) layered closure of the incision, (**i**) initiation of the surgical approach for the transcutaneous implant, selecting an incision that avoids previous dissection areas, following the manufacturer recommendations for Osia™ placement, (**j**) assessment of bone bed regularity; if irregularities are present, bone polishing is performed to achieve a uniform surface, (**k**) placement of the OSI200™ implant, (**l**) layered closure of the incision (l). Images m-p illustrate a separate case of transition surgery using the BI300™ implant from BAHA Connect™ (in this case, a dormant implant), (**m**) the patient experienced pedestal dislodgement, leading to secondary intention wound closure. After surgical field preparation and marking, an incision was made, and skin and subcutaneous tissue were resected over the percutaneous implant site, (**n**) the current BI300™ implant was exposed, and it was decided that the dormant implant was to be used, (**o**) a homogeneous bone bed was confirmed, followed by OSI200™ implant placement, (**p**) the final surgical outcome is shown after layered closure of the incision

The transition surgery was performed under general anesthesia and involved the removal of the percutaneous implant’s abutment (when present) and simultaneous placement of the transcutaneous implant, all as a part of a single-stage surgery. The surgical protocol followed a systematic approach, as outlined in Fig. 2. Sound processor fitting was performed during three to four weeks post-surgery. As depicted in Fig. 2(m-p), some cases involved surgical variations, such as the use of a single incision and the application of the same BI300™ implant when the patient had a specific percutaneous implant placement.

### Statistical analysis

Descriptive analyses were conducted for quantitative variables including interval, proportional, and ordinal data, while frequency distributions were used for nominal variables. Comparative analyses were performed using parametric or non-parametric tests, depending on the distribution and characteristics of the data. Clinical safety outcomes were reported as complication frequencies. Differences in hearing performance before and after transition surgery were assessed by using repeated measures t-tests. Post intervention QoL were analyzed based on the total score and subdomains of the GBI instrument. Statistical significance was set at *p* < 0.05. All analyses were conducted using SPSS version 11.5 (SPSS, Inc., Chicago, IL, USA).

### Ethical considerations

This study was conducted in accordance with the ethical principles outlined in the Declaration of Helsinki [12] and the Colombian Resolution 8430 of 1993 for research involving human subjects [13]. Ethical approval was granted by the Institutional Ethics and Research Committee (CEIFUS 691 − 20, act No. 019–20). To ensure patient confidentiality and privacy, no identifiable personal data were recorded, adhering to national regulatory requirements.

## Results

During the study period, 157 ears (referred to as cases) were scheduled for surgery involving an active transcutaneous BCI. Of these, 23 cases (including five bilateral surgeries) in 18 patients undergoing transition surgeries were eligible and included in the study. The surgical and clinical safety results are presented for two groups: the CHL/MHL group (*n* = 11 cases in *n* = 9 patients including two bilateral surgeries), only consisting of patient with these type of hearing loss, and the Total group, (*n* = 23 cases in *n* = 18 patients) which included the CHL/MHL group and patients with SSD, patients of any age, including those with neurological or cognitive impairment, i.e., all 23 cases.

**Fig. 3:**
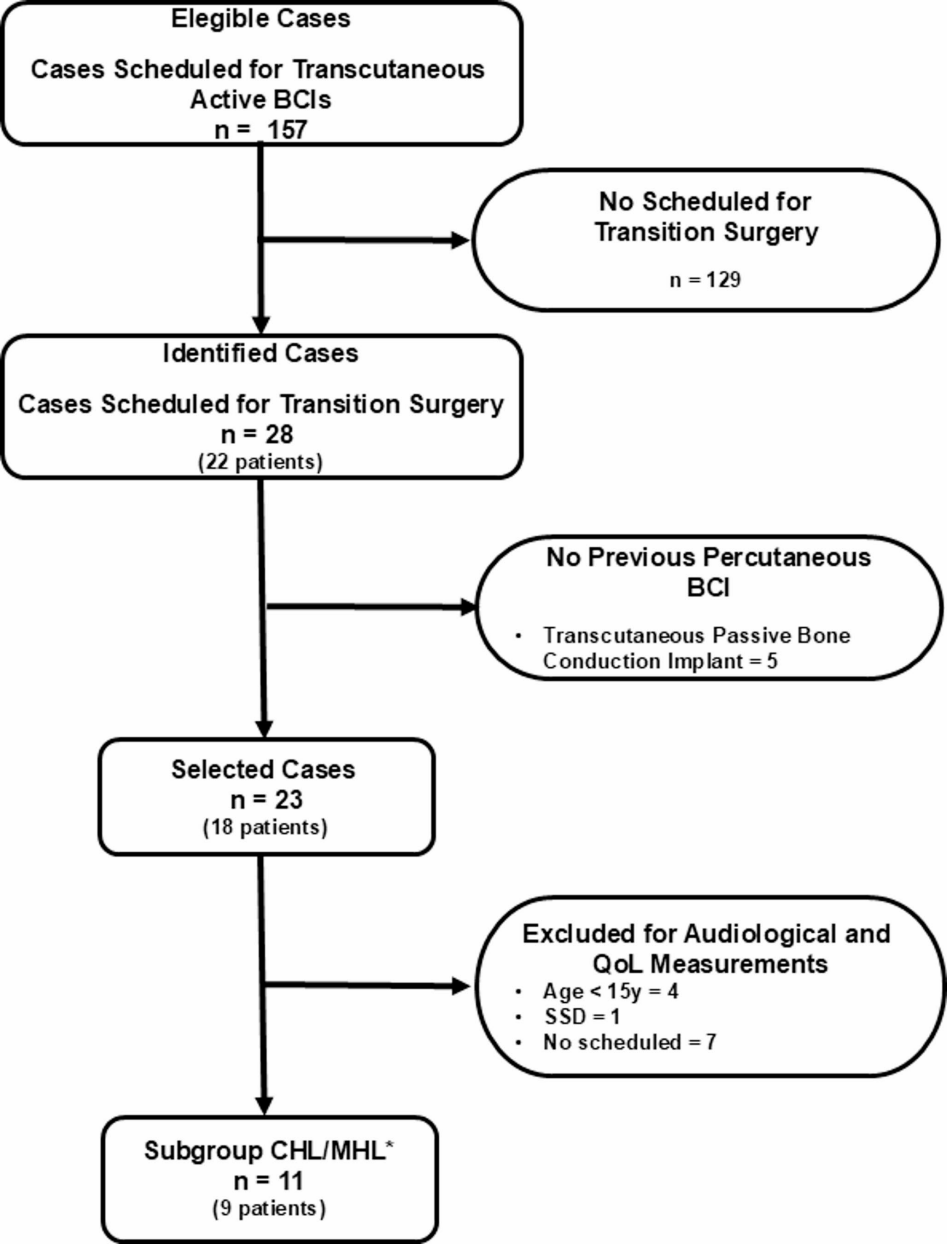
Flowchart of patients included in the study * Comparative audiological analysis between preoperative versus postoperative outcomes (HINT) and measurements of change in QoL (GBI) BCI: Bone conduction implant. QoL: Quality of life. CHL: Conductive hearing loss. MHL: Mixed hearing loss. HINT: Hear In Noise Test. GBI: Glasgow Benefit Inventory

Audiological changes and QoL outcomes post the transition surgery are only presented for the CHL/MHL group. A flowchart detailing the included cases at each stage of the study is provided in Fig. 3.

### Sociodemographic and clinical characteristics

Demographics and clinical characteristics of the two groups are presented separately due to their differing outcomes, ensuring that data is available for consolidation studies, such as meta-analyses. Patients in the CHL/MHL group had a mean age of 39.02 (± 14.59) years. COM was the most common etiology of hearing loss in this group, (54.5%). A little more than half of the patients presented with MHL (54.4%). The mean duration of percutaneous BCI use in this group was 10.2 ± 3.0 years. For the total group the mean age was 33.75 years (± 17.07), and the most common etiology of hearing loss was microtia with external auditory canal atresia/stenosis (65.2%). Sequels of COM accounted for the second most frequent etiology (30.4%). In the total group CHL was the common type of hearing loss (60.8%). The total group had a mean percutaneous BCI use duration of 9.7 (± 2.7 years). The demographic and audiological characteristics of the CHL/MHL group and the overall total group are detailed in Table 1.

**Table 1:** Demographic and clinical characteristics of patients undergoing transition from percutaneous to active transcutaneous BCIs, *n* = 23

Variable	CHL/MHL group *n* = 11 (9 patients)	Total group *n* = 23 (18 patients)
Age at transition surgery (years)* ±SD	39.02 ± 14.59	33.75 ± 17.07
Gender*		
Female	5 (56%)	9 (50%)
Male	4 (44%)	9 (50%)
Side of surgery**		
Right	7 (63.6%)	14 (60.9%)
Left	4 (36.4%)	9 (39.1%)
Type of Hearing Loss**		
Conductive Hearing Loss	5 (45.5%)	15 (65.2%)
Mixed Hearing Loss	6 (54.5%)	7 (30.4%)
Single-Sided Deafness	0	1 (4.4%)
Etiology of Hearing Loss**		
Microtia-atresia/EAC stenosis	5 (45.5%)	15 (65.2%)
Chronic Otitis Media	6 (54.5%)	7 (30.4%)
Sudden Deafness	0	1 (4.4%)
Air-conduction PTA (dB)** ±SD	67.39 ± 7.57	65.40 ± 8.14‡
Bone-conduction PTA (dB)** ±SD	22.05 ± 5.92	20.00 ± 8.35‡
GAP (dB) implanted ear** ±SD	45.35 ± 9.24	45.40 ± 7.16‡
Maximum skin reaction (Holgers Scale)**		
0	0	0
1	0	0
2	3 (27.3%)	4 (17.4%)
3	6 (54.5%)	10 (43.5%)
4	2 (18.2%)	9 (39.1%)
Duration of percutaneous implant use before transition (months)** ±SD	121.9 ± 36.1	115.9 ± 32.3

CHL/MHL group: Only patients with CHL/MHL

Total group: The CHL/MHL group, patients with SSD, and eligible patients of any age, including those with neurological or cognitive impairment

* Values calculated with the number of patients

** Values calculated with the number of cases

‡ Values calculated for 22 cases (SSD case excluded)

*CHL* Conductive Hearing Loss, *MHL* Mixed Hearing Loss, *SD* Standard Deviation, *PTA* Pure Tone Average (0.5–3 kHz), *dB* Decibels, GAP = PTA air conduction– PTA bone conduction, *EAC* External Auditory Canal

### Intraoperative findings

Interoperative findings for the full cohort, i.e., the total group (*n* = 23 ears), showed that most ears operated on (*n* = 19, 82.6%) had a new incision made for the placement of the active transcutaneous BCI, separate from the percutaneous implant scar. In three cases (13%) the abutment of the percutaneous implant had been removed more than one year before the transition surgery and the site had healed by secondary intention. In one case the position of the previous percutaneous implant did not provide sufficient separation between the incision sites, necessitating the use of the existing scar from the percutaneous implant for the new incision. A new osseointegrated implant was used to anchor the transcutaneous BCI in all cases except one, where a pre-existing inactive osseointegrated implant (dormant implant) was used. None of the cases require subcutaneous tissue reduction in the coil area. In four cases (17.4%), where initial flap thickness exceeded 9 mm, the coil was placed superficially to the temporal muscle/fascia, reducing the flap thickness < 8 mm. This approach adhered to the manufacturer’s recommendation of maintaining a tissue thickness < 9 mm between the coil and sound processor to ensure optimal information transmission and secure processor retention.

In 20 cases (87%) a 4 mm osseointegrated implant was used, while the remaining three cases (13%), including the one with the previously implanted device, utilized a 3 mm implant. In most cases (90.9%) which involved utilizing a new osseointegrated implant, it was positioned at the initial drilling site (with a thickness ≥ 3 mm).

In two cases, the implant site required adjustments. One case involved a patient with a skull thickness < 3 mm (in a 12-year-old using a 3 mm implant), while the other case involved a laceration of the sigmoid sinus during the BI300™ bed drilling. To manage the bleeding, a temporal fascia graft was placed in the drilled hole and covered with bone wax under pressure. The implant was then repositioned to an alternate site, a new hole was drilled, and the procedure continued without further complications. Neither of the patients exhibited symptoms related to the event during follow-up.

### Postsurgical follow-up for clinical safety outcomes

**Fig. 4:**
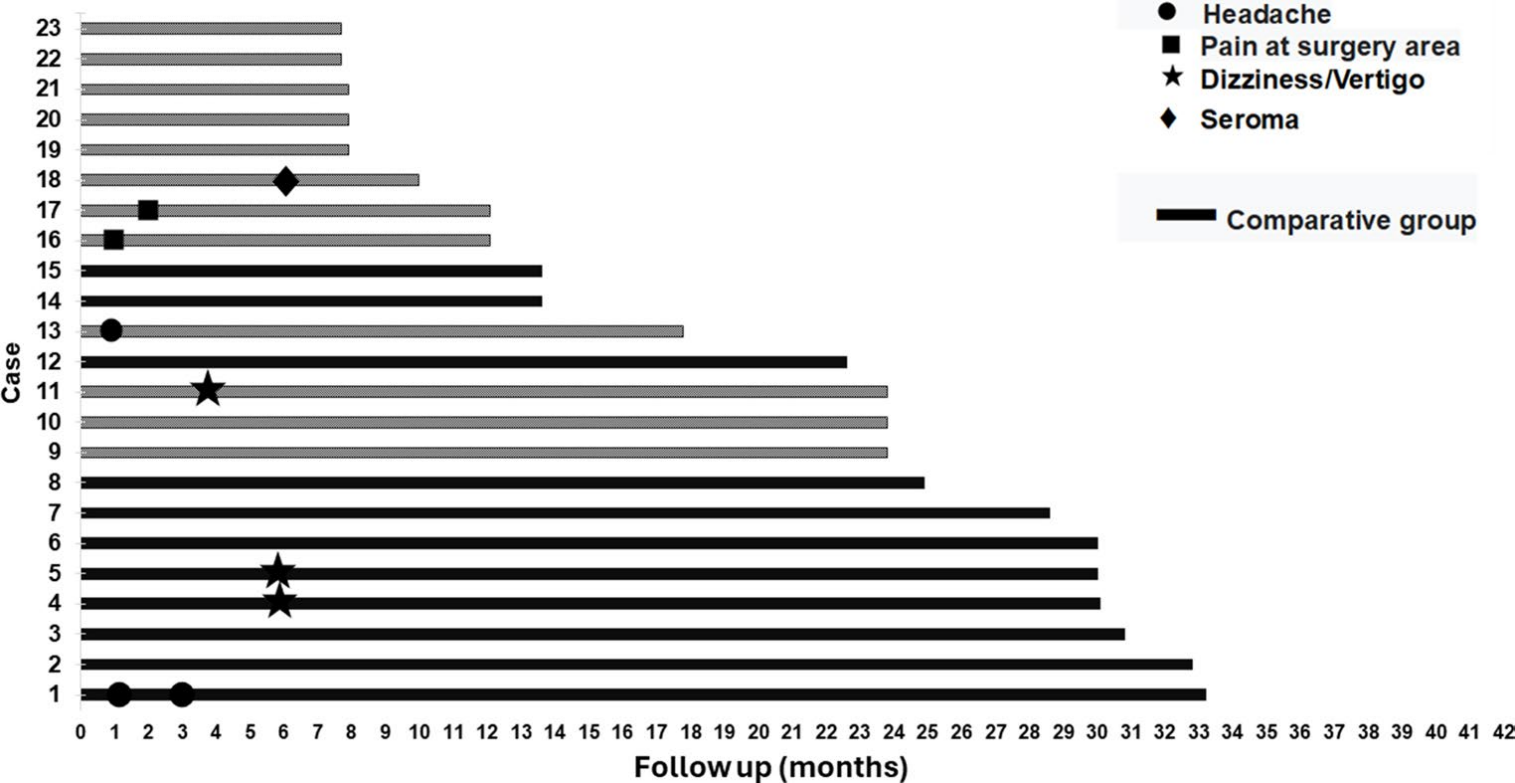
Patient follow-up of study and clinical events during this period

The mean post-surgical follow-up duration for the total group was 19.7 ± 9.4 months (range: 7.7–33.2) and included 113 observation points. No major complications, such as extrusion, surgical site infection, or the need for revision surgery, were reported (Fig. 4). Minor complications occurred in 7.9% of the observations, including dizziness/vertigo in three cases (2.7%), headache in three cases (2.7%), pain at the surgical site in two cases (1.8%), and seroma in one case (0.9%). If the three cases of dizziness/vertigo are excluded, which can be attributed to the patients’ underlying conditions (sudden deafness and COM) [14, 15], the incidence of minor events decreases to 5.3% (6/113). The only case of seroma occurred in a patient who underwent bilateral transition surgery, developing on the right side six months after surgery. Management included needle aspiration, compressive dressing, and a reduction in processor magnet strength, resulting in complete resolution without the need for additional intervention.

### Audiological outcomes for the CHL/MHL group

Hearing performance with the percutaneous implant for the CHL/MHL group (*n* = 9 patients and *n* = 11 ears) prior to transition surgery was favorable, with an unaided PTA of 65.09 ± 10.39dB and an aided PTA in the free field of 29.00 ± 7.01 dB, resulting in a FG of 36.09 ± 11.95dB. This difference was statistically significant (*p* < 0.001).

Audiometric measurements post transition surgery were conducted on average 7.22 ± 2.16 months after fitting the sound processor. Comparative analysis of the HINT outcomes before and after transition surgery showed no statistically significant differences between the percutaneous and transcutaneous implants in either fixed noise (*p* = 0.99) or adaptive noise (*p* = 0.617) conditions, as detailed in Table 2.

**Table 2:** Audiological outcomes in patients transitioning from percutaneous to active transcutaneous BCIs in patients with CHL/MHL, *n* = 11

Measurement	Percutaneous BCI (Mean ± SD)	Transcutaneous BCI (Mean ± SD)	Difference (Mean ± SD) *p*-value*
HINT fixed noise (SNR = 0) Speech = 65dB/Noise = 65dB (% correct) x ± SD	88.485 ± 6.46	88.475 ± 6.73	−0.011 ± 9.643 ***p*** **= 0.99**
HINT adaptive noise Speech = 65dB/Noise = 60dB (SNR, dB) x ± SD	−4.055 ± 1.14	−4.291 ± 1.51	0.236 ± 1.520 ***p*** **= 0.617**

*Repeated measures t-test

*CHL* Conductive Hearing Loss, *MHL* Mixed Hearing Loss, *HINT* Hearing in Noise Test, *SNR* Signal-to-Noise Ratio, *SD* Standard Deviation, *dB* Decibels

### Post-surgical QoL outcomes in the CHL/MHL group

Quality of life outcomes in the CHL/MHL group were assessed following transition surgery using the GBI. The results demonstrated a moderate overall positive impact, with a mean total score of + 27.0 ± 33.9 (range: −25.0 to + 61.1). Subdomain analysis revealed the greatest improvement in general health status (+ 29.9 ± 33.1,range: −20.8 to + 62.5), and social support (+ 31.8 ± 36.1, range: −16.7 to + 83.3), while the impact on physical well-being was more modest (+ 10.6 ± 40.3,range: −50.0 to + 66.7). These findings indicate a generally favorable, albeit variable, improvement in QoL, with the most pronounced benefits observed for general health and social support.

## Discussion

The findings of this study suggest that transitioning from a percutaneous BCI to a transcutaneous active BCI can be safely performed as a single-stage procedure. This conclusion is based on 23 surgeries, with a mean follow-up of 19.7 months (range:7.7 to 33.2 months). No major complications were reported, and minor complications occurred in 7.9% of the cases (Fig. 4). Notably, if excluding the three cases of dizziness/vertigo, likely attributable to preexisting conditions, such as, COM or sudden deafness [14, 15], the incidence of minor complications is reduced to 5.3%.

These findings support a favorable clinical safety profile for transition surgery, particularly considering the inherent challenge of moving from a contaminated surgical field, due to bacterial colonization around the percutaneous implant abutment, to a clean surgical field for active transcutaneous BCI placement. Given that a contaminated field is a known risk factor for surgical site infections [16], achieving this transition in a single-stage procedure reinforces the safety and feasibility of the approach. A meticulous surgical plan is critical to minimizing infection risk, with key steps including the eradication of bacterial colonies around the abutment and the careful isolation of the dissection field for both implant removal and replacement (Fig. 2h and i-l). In the context of eradicating bacterial colonies, we emphasize the necessity of resecting all skin and subcutaneous tissues surrounding the osseointegrated implant (Fig. 2d, e, and f). Additionally, thorough soaking and irrigation of the surgical site with a 2% chlorhexidine solution for a minimum of five minutes (Fig. 2g) is recommended to further reduce bacterial load [17, 18]. The 19.7-month follow-up period (range: 7.7–33.2 months) in this study provides an adequate timeframe to assess major postoperative infections associated with transition surgery. However, the potential for late-onset complications, such as biofilm-associated infections [19], remains unknown. As such, while this study provides valuable medium-term data, longer follow-up studies extending up to five years are needed to draw definitive conclusions regarding the long-term infectious risks associated with this surgical transition (Fig. 4).

Additionally, the findings regarding clinical safety highlight the importance of careful surgical planning, particularly when selecting incision site in patients with prior implants. Managing pre-existing scar tissue can present challenges, underscoring the need for a tailored approach to optimize surgical outcomes. This consideration is consistent with observations reported by when Florentine et al. [20], who also emphasized the impact of previous implant scars on surgical planning and execution.

Only one intraoperative complication was observed in the study, which a laceration of the sigmoid sinus. This finding is consistent with previous studies on primary Osia surgeries, where intraoperative complications are generally minor, rare, or non-existent [20–23]. The minor complications associated with transition surgery, including headaches and pain at the surgical site, are common postoperative symptoms, likely attributable to the body’s natural healing response following surgery. The single case of seroma, effectively managed with conservative treatment, may have resulted from the body’s reaction to the implant or minor scalp trauma related to routine device use. The successful resolution suggests that such complications can be effectively controlled with appropriate postoperative care. Overall, the low incidence of minor complications and the absence of major complications reinforce the safety of transition surgery to Osia. However, patients with a history of COM or sudden deafness may require closer postoperative monitoring and preoperative counseling due to their increased risk of experiencing dizziness or vertigo [14, 15].

Before undergoing transition surgery, patients (*n* = 18 patients, *n* = 23 ears) had an average unaided air-conduction PTA = 65.09 ± 10.39 dB, and bone-conduction PTA = 20.00 ± 8.35dB, consistent with moderate-to-severe predominantly conductive hearing loss. Aided by the percutaneous BCI, PTA showed a significant improvement, with an average aided PTA = 29.00 ± 7.01dB. This corresponded to a FG of 36.09 ± 11.95dB (*p* < 0.001), which falls within the clinically significant range of 30–40 dB, indicating a substantial improvement in hearing ability.

For the evaluation of audiological performance and QoL outcomes in the CHL/MH subgroup, the analysis was limited to 11 ears and 9 patients. The study found no significant differences in HINT scores (Table 2) between percutaneous BCIs and active transcutaneous BCIs. This finding may be attributed to the already high performance of the percutaneous device in patients affected by CHL/MHL. Given that CHL primarily affects lower frequencies, the additional gain at higher frequencies provided by the active transcutaneous device may offer limited benefit for this patient group. These findings highlight the importance of comprehensive patient counseling regarding expected hearing outcomes when considering transition surgery from percutaneous to an active transcutaneous BCIs in patients with CHL/MHL.

Conversely, in cases of transition from a passive transcutaneous implant to an active transcutaneous system, a comparative study of the Baha Attract and Osia systems following transition surgery demonstrated that the Osia system consistently outperforms the Baha Attract across all test frequencies, with particularly notable improvements in speech audiometry [24].

Quality of life outcomes following transition surgery, as assessed by the GBI in the CHL/MHL group, showed a moderate positive improvement, with an average total score of + 27.0 ± 33.9 (range: −25.0 to + 61.1). While these findings suggest a general improvement in well-being, individual responses varied considerably. The general health domain showed a moderate improvement, with an average score of + 29.9 ± 33.1 (range: −20.8 to + 62.5), indicating better overall health. The social support domain showed the highest average score of + 31.8 ± 36.1 (range: −16.7 to + 83.3), suggesting improved social interactions and support, likely attributable to better communication, though the with substantial variability. In contrast, the physical well-being domain had the lowest average score of + 10.6 ± 40.3 (range: −50.0 to + 66.7), reflecting only modest improvements in physical health, with some patients reporting postoperative challenges.

Overall, transition surgery generally improved QoL, particularly in the domains of general health and social support, while benefits in physical well-being were more variable and limited. This variability underscores the need for personalized patient care and the importance of setting realistic expectations. However, it is important to acknowledge that the GBI includes questions that may not be directly related to the surgical intervention itself, such as “Since your operation, are there more or fewer people who really care for you?” and “Since your operation, do you catch colds or infections more or less often?”. As a result, some variability in GBI responses may be influenced by external factors unrelated to the surgery. The broad and nonspecific nature of the GBI may reflect general life circumstances rather than accurately capturing the specific effects of the transition surgery, contributing to uncertainty in the interpretation of its effect.

The study has several strengths and limitations. One of its key strengths is the prospective design and the rigorous clinical safety monitoring conducted in a representative patient group with medium-term follow-up. Additionally, the study employed comprehensive audiological assessments for the CHL/MHL group, including HINT, ensuring a robust evaluation of hearing outcomes. A limitation of this study is the small sample size (*n* = 9) for audiological and quality of life outcomes, which restricts the statistical power and generalizability of these findings. Despite this limitation, the data provide valuable preliminary insights that can guide future research with larger cohorts. Additionally, the absence of a control group restricts the ability to directly compare outcomes and definitively assess efficacy. However, the main objective of this study was to demonstrate the clinical and surgical safety of transition surgery, rather than to focus on audiological improvement. Another notable limitation is the use of the GBI, a general QoL assessment tool. While the GBI effectively measures treatment-related changes, it may not fully capture the specific challenges and needs of individual hearing loss. Furthermore, the observed variability in QoL outcomes, particularly in the physical well-being domain, suggests significant inter-individual differences in patient experiences, emphasizing the need for a more tailored approach to assessing postoperative QoL in this population. Although the GBI is designed solely for post-intervention use and therefore does not provide a preoperative baseline for comparison, it is a valid instrument for QoL assessment in otolaryngology [10, 11]. Future research should address these limitations by increasing sample sizes, extending the follow-up period to at least 5 years, and further explore the variability in patient experiences for QoL. This could be achieved through the use of both generic and disease specific QoL questionnaires administered pre- and post-surgery, allowing for a more comprehensive evaluation of patient-reported outcomes. Such an approach would help validate and refine the findings of this study, ultimately contributing to a more nuanced understanding of the long-term benefits and challenges associated with transition surgery from a percutaneous to an active transcutaneous BCI.

## Conclusion

The study demonstrates that the transition surgery from a percutaneous to an active transcutaneous BCI in a single-stage procedure is safe, with no major complications and minimal minor adverse events. Audiological outcomes with active transcutaneous BCIs were comparable to those of percutaneous implants, preserving hearing performance. Additionally, in the CHL/MHL group improvements were observed in QoL particularly in general health and social support. These findings provide valuable clinical insights for guiding patient counseling and optimizing post-operative care.

## Electronic supplementary material

Below is the link to the electronic supplementary material.

Supplementary Material 1
