# Supplementary material for: Clinical and surgical safety in patients transitioning from percutaneous to transcutaneous active bone conduction implants
Source: Eur Arch Otorhinolaryngol. 2025 Aug 8;283(1):69–79. doi: 10.1007/s00405-025-09610-7 (PMC12904925; doi:10.1007/s00405-025-09610-7)
Supplement: Supplementary file 1 — Supplementary Material 1 [file 405_2025_9610_MOESM1_ESM.pdf]

demograp	gender	s_patient_birth	s_surgery_date	grupcompa	etiology	s_incision_	incisionnev
1	2	8-nov-65	9-dic-20	1	2	Holgers 2	1
1	1	11-feb-82	21-dic-20	1	1	Holgers 3	1
1	2	15-jul-80	17-feb-21	1	1	Holgers 2	1
1	1	12-sep-88	15-mar-21	1	2	Holgers 3	1
2	1	12-sep-88	15-mar-21	1	2	Holgers 3	1
1	1	3-abr-06	24-abr-21	1	1	Holgers 4	2
1	2	1-ene-66	14-ago-21	1	2	Holgers 4	1
1	2	13-ago-08	15-sep-21	2	1	Holgers 4	1
2	2	13-ago-08	15-sep-21	2	1	Holgers 3	1
1	2	16-jun-10	15-sep-21	2	1	Holgers 4	1
1	2	15-nov-59	16-mar-22	2	3	Holgers 3	1
1	1	30-mar-03	20-jul-22	1	1	Holgers 3	1
2	1	30-mar-03	20-jul-22	1	1	Holgers 2	1
1	1	1-jun-10	3-sep-22	2	1	Holgers 4	1
1	1	30-jun-75	3-sep-22	2	2	Holgers 4	1
1	1	5-nov-03	4-nov-22	2	1	Holgers 4	1
1	2	13-dic-82	5-ene-23	2	1	Holgers 3	1
2	2	13-dic-82	5-ene-23	2	1	Holgers 3	1
1	1	8-nov-07	6-ene-23	2	1	Holgers 2	2
1	1	21-feb-87	12-ene-23	2	1	Holgers 4	2
2	1	21-feb-87	12-ene-23	2	1	Holgers 4	2
1	2	26-mar-78	10-mar-21	1	2	Holgers 3	1
1	2	15-nov-69	22-oct-21	1	2	Holgers 3	1

surgeyage	s_patient_	datesgconnec	connecttin	followdatecutc	followupm	s_loss_typ	compintra	complipri
55,1	Derecho	14-sep-13	88,1	31-ago-23	33,2	3	0	4
38,9	Derecho	1-jul-10	127,5	31-ago-23	32,8	2	0	0
40,6	Derecho	1-jul-11	117,3	31-ago-23	30,8	3	0	0
32,5	Izquierdo	8-feb-07	171,6	31-ago-23	30,0	3	0	0
32,5	Derecho	8-feb-07	171,6	31-ago-23	30,0	2	0	0
15,1	Izquierdo	3-may-13	97,1	31-ago-23	28,6	3	0	0
55,7	Derecho	11-ago-14	85,3	31-ago-23	24,9	2	0	0
13,1	Izquierdo	4-jun-13	100,8	31-ago-23	23,8	2	0	0
13,1	Derecho	4-jun-13	100,8	31-ago-23	23,8	2	0	0
11,3	Derecho	17-sep-12	109,5	31-ago-23	23,8	2	0	0
62,4	Izquierdo	18-ago-15	80,1	31-ago-23	17,8	#¡NULO!	3	0
19,3	Izquierdo	4-mar-13	114,2	31-ago-23	13,6	2	0	0
19,3	Derecho	4-mar-13	114,2	31-ago-23	13,6	2	0	4
12,3	Derecho	24-ago-17	61,2	31-ago-23	12,1	2	0	0
47,2	Derecho	3-nov-15	83,2	31-ago-23	12,1	3	0	0
19,0	Derecho	8-ene-12	131,8	31-ago-23	10,0	2	0	2
40,1	Izquierdo	23-ago-10	150,6	31-ago-23	7,9	2	0	0
40,1	Derecho	23-ago-10	150,6	31-ago-23	7,9	2	0	0
15,2	Izquierdo	3-may-15	93,5	31-ago-23	7,9	3	0	0
35,9	Izquierdo	23-mar-12	131,6	31-ago-23	7,7	2	0	0
35,9	Derecho	23-mar-12	131,6	31-ago-23	7,7	2	0	0
43,0	Derecho	2-oct-14	78,4	31-ago-23	30,1	3	0	0
51,0	Izquierdo	14-may-07	175,8	31-ago-23	22,6	3	0	0

compli1a3	compli3a6	compli6a1	compli12a	compli18a	compli24a	compli30a	followupn	s_users_id	s_previous
4	0	0	0	0	0	#¡NULO!		#¡NULO!	1
0	0	0	0	0	0	0		#¡NULO!	1
0	0	0	0	0	0	#¡NULO!		#¡NULO!	1
0	0	5	0	0	#¡NULO!	#¡NULO!		#¡NULO!	1
0	0	5	0	0	#¡NULO!	#¡NULO!		#¡NULO!	1
0	0	0	0	0	#¡NULO!	#¡NULO!	No se hizo	#¡NULO!	1
0	0	0	0	0	#¡NULO!	#¡NULO!		#¡NULO!	1
0	0	0	0	0	#¡NULO!	#¡NULO!		#¡NULO!	1
0	0	0	0	0	#¡NULO!	#¡NULO!		#¡NULO!	1
0	0	0	0	#¡NULO!	#¡NULO!	#¡NULO!		#¡NULO!	1
5	0	0	0	#¡NULO!	#¡NULO!	#¡NULO!	Presentó s	#¡NULO!	1
0	0	0	#¡NULO!	#¡NULO!	#¡NULO!	#¡NULO!		#¡NULO!	1
0	0	0	#¡NULO!	#¡NULO!	#¡NULO!	#¡NULO!		#¡NULO!	1
0	0	0	#¡NULO!	#¡NULO!	#¡NULO!	#¡NULO!	Requirió fr	#¡NULO!	1
0	0	0	#¡NULO!	#¡NULO!	#¡NULO!	#¡NULO!		#¡NULO!	1
0	0	#¡NULO!	#¡NULO!	#¡NULO!	#¡NULO!	#¡NULO!		#¡NULO!	1
2	0	#¡NULO!	#¡NULO!	#¡NULO!	#¡NULO!	#¡NULO!		#¡NULO!	1
0	6	#¡NULO!	#¡NULO!	#¡NULO!	#¡NULO!	#¡NULO!	Drenaje de	#¡NULO!	1
0	0	#¡NULO!	#¡NULO!	#¡NULO!	#¡NULO!	#¡NULO!	No incisión	#¡NULO!	1
0	0	#¡NULO!	#¡NULO!	#¡NULO!	#¡NULO!	#¡NULO!	No incisión	#¡NULO!	1
0	0	#¡NULO!	#¡NULO!	#¡NULO!	#¡NULO!	#¡NULO!	No incisión	#¡NULO!	1
0	0	0	0	0	0	#¡NULO!		#¡NULO!	1
0	0	0	0	0	#¡NULO!	#¡NULO!		#¡NULO!	1

s_previous	s_transitio	s_transitio	s_thicknes	s_thicknes	s_size	s_va125	s_va250	s_va500	s_va1000
	Si	BAHA CON	No se hizo	5	4mm	No Testad	70	70	70
	Si	BAHA CON	No se hizo	0	4mm	60	60	65	65
	Si	BAHA CON	No se hizo	0	4mm	90	90	95	80
	Si	CONNECT	No se hizo	7	4mm	55	60	60	60
	Si	CONNECT	No se hizo	0	4mm	65	70	70	70
	Si	BAHA CON	No se hizo	6	3mm	65	65	65	65
	Si	BAHA CON	No se hizo	0	4mm	65	60	55	55
	Si	Baha Conn	No se hizo	0	4mm	No Testad	60	55	60
	Si	Baha Conn	No se hizo	0	4mm	No Testad	60	60	60
	Si	BahaConn	No se hizo	0	4mm	No Testad	60	65	60
	Si	Baha	No se hizo	7	4mm	No Testad	110	110	110
	Si	BAHA CON	No se hizo	0	4mm	80	80	75	65
	Si	BAHA CON	No se hizo	0	4mm	80	80	75	70
	Si	BAHA CON	No se hizo	0	3mm	50	45	50	50
	Si	BAHA CON	No se hizo	0	4mm	60	60	65	80
	Si	BAHA CON	No se hizo	0	4mm	80	70	65	65
	Si	baha attra	No se hizo	0	4mm	No Testad	70	70	70
	Si	baha attra	No se hizo	0	4mm	No Testad	85	75	75
	Si	CONNECT	No se hizo	0	4mm	100	85	75	60
	Si	Connect	No se hizo	0	4mm	70	70	65	65
	Si	Connect	No se hizo	0	3mm	70	70	70	70
	Si	Connect	No se hizo	#¡NULO!	4mm	80	70	65	70
	Si	Connect	No se hizo	0	4mm	No Testad	90	85	85

s_va2000	s_va3000	s_va4000	s_va6000	s_va8000	s_vo125	s_vo250	s_vo500	s_vo1000	s_vo2000
55	55	60	55	50	No Testad	20	30	25	35
70	70	70	75	70	15	15	15	20	20
75	70	70	75	80	25	25	25	20	25
65	65	70	75	75	10	15	15	20	25
75	80	70	75	75	10	10	15	15	25
65	55	55	60	65	20	20	20	20	25
50	50	60	65	95	10	10	25	15	20
60	55	65	65	No Testad	No Testad	10	15	20	10
55	60	65	60	No Testad	No Testad	10	15	20	10
60	65	65	65	No Testad	No Testad	10	15	15	10
110	110	110	110	No Testad	No Testad	70	70	70	70
70	60	55	70	55	5	5	15	10	20
65	65	70	65	70	5	5	10	10	20
60	60	65	65	50	10	10	10	10	10
95	105	105	95	95	10	#¡NULO!	40	40	55
55	50	70	65	65	15	15	10	10	10
60	55	60	55	65	No Testad	5	10	15	20
65	65	60	65	70	No Testad	5	15	10	20
55	55	50	55	65	15	20	10	25	25
55	55	45	55	55	10	10	15	15	20
60	55	60	60	45	15	15	20	20	5
65	70	70	75	75	20	25	35	35	30
70	65	65	85	No Testad	No Testad	15	30	25	30

s_vo3000	s_vo4000	s_vo6000	s_vo8000	ptavaerea	ptavosea	gap	f3_speak_	f3_speak_	f3_speak_
25	25	No Testad	No Testad	62,5	28,8	33,8	Si	SRT	
20	20	No Testad	No Testad	67,5	18,8	48,8	Si	Discriminación	
20	25	No Testad	No Testad	80,0	22,5	57,5	Si	Discriminación	
25	20	No Testad	No Testad	62,5	21,3	41,3	Si	Sonidos de	Sonidos de
25	20	No Testad	No Testad	73,8	20,0	53,8	Si	Sonidos de	Sonidos de
20	20	No Testad	No Testad	62,5	21,3	41,3	Si	Sonidos de	Sonidos de
20	20	No Testad	No Testad	52,5	20,0	32,5	Si	Discrimina	Discrimina
10	15	No Testad	No Testad	57,5	13,8	43,8	Si	Discrimina	Discrimina
10	15	No Testad	No Testad	58,8	13,8	45,0	Si	Discrimina	Discrimina
10	15	No Testad	No Testad	62,5	12,5	50,0	Si	SRT, Sonid	SRT, Sonid
70	70	No Testad	No Testad	#¡NULO!	#¡NULO!	#¡NULO!	Si	Sonidos de	Sonidos de
15	10	No Testad	No Testad	67,5	15,0	52,5	Si	Sonidos de	Sonidos de
15	10	No Testad	No Testad	68,8	13,8	55,0	Si	Sonidos de	Sonidos de
10	10	No Testad	No Testad	55,0	10,0	45,0	Si	Sonidos de	Sonidos de
50	45	No Testad	No Testad	86,3	46,3	40,0	Si	Discrimina	Sonidos de
15	20	No Testad	No Testad	58,8	11,3	47,5	Si	Discrimina	Discrimina
25	10	No Testad	No Testad	63,8	17,5	46,3	Si	Sonidos de	Sonidos de
20	15	No Testad	No Testad	70,0	16,3	53,8	Si	Sonidos de	Sonidos de
30	35	No Testad	No Testad	61,3	22,5	38,8	Si	Sonidos de	Sonidos de
30	15	No Testad	No Testad	60,0	20,0	40,0	Si	Discrimina	Discrimina
10	10	No Testad	No Testad	63,8	13,8	50,0	Si	Discrimina	Discrimina
25	25	25	25	67,5	33,8	33,8	Si	Discriminación	
25	25	No Testad	No Testad	76,3	27,5	48,8	Si	Discrimina	Discrimina

f3_results_	f3_audiometry_dat	f3_poweron_date	f3_consultati	f3_magnet	f3_magnet	f3_va250	f3_va500
	6-abr-21	2-feb-21	6-abr-21	No	#¡NULO!	25	25
	11-may-21	9-feb-21	11-may-21	No	#¡NULO!	45	40
	29-jun-21	18-mar-21	29-jun-21	No	#¡NULO!	20	15
1	4-sep-21	22-abr-21	4-sep-21	No	#¡NULO!	35	25
1	4-sep-21	22-abr-21	4-sep-21	No	#¡NULO!	35	35
100% A 50	4-sep-21	27-may-21	4-sep-21	No	#¡NULO!	No Testad	No Testad
discrimina	18-dic-21	16-sep-21	18-dic-21	No	#¡NULO!	40	35
discrimina	15-ene-22	19-ene-22	19-ene-22	Si	3	45	50
DISCRIMIN	15-ene-22	17-feb-22	19-oct-21	No	#¡NULO!	45	50
!00% a 50	8-feb-22	14-oct-21	8-feb-22	No	#¡NULO!	20	20
100% A VC	18-ago-22	14-abr-22	18-ago-22	No	#¡NULO!	55	55
1	3-ene-23	16-ago-22	3-ene-23	No	#¡NULO!	40	35
1	3-ene-23	2-ago-22	3-ene-23	No	#¡NULO!	35	25
1	30-dic-22	30-sep-22	30-dic-22	No	#¡NULO!	25	30
IDENTIFICA	19-ene-23	30-sep-22	19-ene-23	No	#¡NULO!	75	80
DISCRIMIN	21-dic-22	3-dic-22	21-dic-22	No	#¡NULO!	No Testad	No Testad
100% A VC	10-may-23	3-feb-23	10-may-23	Si	3	45	35
100% A VC	11-may-23	3-feb-23	10-may-23	Si	3	45	40
100% A VC	4-jul-23	7-feb-23	4-jul-23	No	#¡NULO!	30	25
DISCRIMIN	19-abr-23	15-feb-23	19-abr-23	Si	4	35	25
DISCRIMIN	19-abr-23	15-feb-23	19-abr-23	Si	4	35	25
	#NULL!	#NULL!	#NULL!	No	#¡NULO!	40	25
DISCRIMIN	#NULL!	#NULL!	#NULL!	Si	3	20	35

f3_va1000	f3_va2000	f3_va3000	f3_va4000	f3_va6000	f3_va8000	f12_speak	f12_speak	f12_result	f12_speak
20	20	25	15	20	No Testad	Si	Sonidos de	1	Si
40	35	35	40	25	25	Si	Discrimina	DISCRIMIN	Si
15	20	20	20	15	No Testad	Si	Discrimina	discrimina	Si
25	30	25	35	20	35	Si	Sonidos de	1	Si
35	25	40	35	20	50	Si	Sonidos de	1	Si
No Testad	No Testad	No Testad	No Testad	No Testad	No Testad	Si	SRT, Sonid	1	Si
25	20	25	25	25	No Testad	Si	Discrimina	DISCRIMIN	Si
45	45	50	45	50	No Testad	Si	Discrimina	DISCRIMIN	Si
45	45	50	45	45	50	Si	Discrimina	discrimina	Si
20	20	20	20	No Testad	No Testad	Si	Sonidos de	100% a vo	Si
30	50	40	50	30	No Testad	Si	Sonidos de	100% A vo	Si
30	30	25	25	No Testad	No Testad	Si	Sonidos de	100% a 45	No
25	25	20	25	No Testad	No Testad	Si	Sonidos de	100% a 45	Si
25	25	20	20	No Testad	No Testad	Si	Sonidos de	100% a 45	Si
90	95	95	95	No Testad	No Testad	Si	Sonidos de	100% A 45	Si
No Testad	No Testad	No Testad	No Testad	No Testad	No Testad	Si	Sonidos de	100% A 40	Si
25	25	25	35	30	No Testado				
30	25	25	35	20	No Testado				
20	20	20	25	No Testad	No Testad	Si	Sonidos de	100% a 50	Si
20	15	15	25	15	No Testad	Si	Sonidos de	100% A 50	Si
20	15	20	35	20	No Testad	Si	Sonidos de	100% A 50	Si
25	25	30	30	20	35	Si	Discrimina	DISCRIMIN	Si
30	25	25	25	20	No Testad	Si	Discrimina	DISCRIMIN	Si

f12_audiometry_	f12_poweron	f12_consu	f12_magn	f12_va250	f12_va500	f12_va100	f12_va200	f12_va300
14-feb-22	2-feb-21	14-feb-22	No	20	20	20	25	25
12-ene-22	9-ene-21	########	No	40	25	35	35	25
21-feb-22	18-feb-22	21-feb-22	No	45	45	25	25	20
15-mar-22	22-abr-21	########	No	35	30	30	20	35
15-mar-22	22-abr-21	########	No	25	15	25	30	30
30-abr-22	27-may-21	30-abr-22	No	35	30	20	15	25
24-ago-22	19-ago-22	24-ago-22	No	30	35	25	25	30
17-dic-22	19-oct-21	13-ago-08	Si	20	20	10	10	20
17-dic-22	19-oct-21	13-ago-08	Si	20	20	10	10	20
8-nov-22	14-oct-21	8-nov-22	No	35	30	15	15	25
9-may-23	11-abr-22	9-may-23	No	40	40	25	25	30
16-sep-23	28-ago-22	9-sep-23	No	#¡NULO!	#¡NULO!	#¡NULO!	No Testad	#¡NULO!
9-sep-23	2-ago-22	9-sep-23	No	25	15	15	No Testad	25
18-oct-23	18-oct-23	30-sep-22	No	40	20	30	35	40
4-nov-23	3-sep-22	4-nov-23	No	45	45	40	50	70
30-nov-23	3-dic-22	########	Si	35	25	15	25	15
#NULL!	#NULL!	#NULL!		#¡NULO!	#¡NULO!	#¡NULO!		#¡NULO!
#NULL!	#NULL!	#NULL!		#¡NULO!	#¡NULO!	#¡NULO!		#¡NULO!
26-ene-24	25-sep-23	16-feb-24	No	20	25	20	20	25
15-feb-24	15-feb-23	15-feb-24	No	40	25	20	20	25
15-feb-24	15-feb-23	15-feb-24	No	45	25	20	20	25
#NULL!	#NULL!	#NULL!	No	30	20	20	20	25
#NULL!	#NULL!	#NULL!	No	35	30	20	25	20

f12_va400	f12_va600	c_p1_pre	c_p1_pos	c_p1a	c_p1b	c_p1_extr	c_p2_pre	c_p2_pos	c_p2a
20	25	4	4	5	5		2	2	5
30	20	10	10	5	4		2	2	5
30	20	4	4	5	5		2	2	5
30	20	11	11	5	5		2	2	4
20	No Testad	11	11	5	5		2	2	4
25	25	4	4	5	5		2	2	5
35	20	4	4	4	4		6	6	4
20	10	4	4	5	5		2	2	5
20	10	12	12	5	5		13	13	5
25	15	4	4	4	4		6	6	4
35	25	4	4	4	4		5	5	4
#¡NULO!	No Testad	4	4	4	4		3	3	5
15	15	4	4	4	4		3	3	4
35	35	4	4	4	5		15	15	4
70	70	16	16	4	4	No acercar	4	4	4
15	15	2	2	5	4		4	4	4
#¡NULO!		15	#¡NULO!	#¡NULO!	#¡NULO!		2	#¡NULO!	#¡NULO!
#¡NULO!		15	#¡NULO!	#¡NULO!	#¡NULO!		2	#¡NULO!	#¡NULO!
20	No Testad	4	4	4	4		6	6	4
20	35	2	2	4	4		6	6	4
30	25	4	4	4	4		6	6	4
25	15	1	1	5	5		13	13	5
20	20	2	2	5	5		4	4	5

c_p2b	c_p3_pre	c_p3_pos	c_p3a	c_p3b	c_p3_extra	c_p4_pre	c_p4_pos	c_p4a	c_p4b
5	11	11	4	4		15	15	4	4
3	4	4	5	5		7	7	5	4
5	8	8	5	5		9	9	5	5
4	4	4	4	4		5	5	5	5
4	4	4	4	4		5	5	5	5
5	3	3	5	5		7	7	5	5
4	5	5	3	4		8	8	4	4
5	11	11	5	5		12	12	5	5
5	2	2	5	5		3	3	5	5
4	7	7	5	5		5	5	5	5
5	11	11	4	5		2	2	4	5
4	2	2	5	4		11	11	5	4
5	2	2	5	4		11	11	5	4
5	11	11	5	5		6	6	5	5
4	11	11	5	5		15	15	5	5
3	8	8	4	4		11	11	5	5
#¡NULO!	8	#¡NULO!	#¡NULO!	#¡NULO!		4	#¡NULO!	#¡NULO!	#¡NULO!
#¡NULO!	8	#¡NULO!	#¡NULO!	#¡NULO!		4	#¡NULO!	#¡NULO!	#¡NULO!
4	2	2	3	3		9	9	5	5
5	7	7	4	4		8	8	5	5
4	2	2	4	4		14	14	5	5
5	3	3	4	5		5	5	5	5
5	6	6	5	5		8	8	5	5

c_p5_pre	c_p5_pos	c_p5a	c_p5b	c_p5_extra
6	6	4	4	
6	6	5	5	
10	10	5	5	
9	9	5	5	
9	9	5	5	
16	16	5	5	ESCUCHAR MUSICA
11	11	4	4	
13	13	5	5	
4	4	5	5	
11	11	5	5	
15	15	4	5	
15	15	5	4	
15	15	5	5	
5	5	5	5	
16	16	5	5	Que se me quite el TINNITUS.
9	9	5	5	
5	#¡NULO!	#¡NULO!	#¡NULO!	
5	#¡NULO!	#¡NULO!	#¡NULO!	
15	15	5	5	
5	5	5	5	
11	11	5	5	
12	12	5	5	
15	15	5	5	
